# A Standardized Protocol for Efficient and Reliable Quality Control of Brain Registration in Functional MRI Studies

**DOI:** 10.3389/fninf.2020.00007

**Published:** 2020-02-28

**Authors:** Yassine Benhajali, AmanPreet Badhwar, Helen Spiers, Sebastian Urchs, Jonathan Armoza, Thomas Ong, Daniel Pérusse, Pierre Bellec

**Affiliations:** ^1^Département d’Anthropologie, Université de Montréal, Montreal, QC, Canada; ^2^Centre de Recherche de l’Institut Universitaire de Gériatrie de Montréal (CRIUGM), Montreal, QC, Canada; ^3^The Zooniverse, Department of Physics, University of Oxford, Oxford, United Kingdom; ^4^Electron Microscopy Science Technology Platform, The Francis Crick Institute, London, United Kingdom; ^5^Integrated Program in Neuroscience, Montreal Neurological Institute, Montreal, QC, Canada; ^6^English Department, New York University, New York, NY, United States; ^7^Jewish General Hospital, Department of Radiology, McGill University, Montreal, QC, Canada; ^8^Département de Psychologie, Université de Montréal, Montreal, QC, Canada

**Keywords:** quality control, fMRI, brain registration, crowdsourcing, visual inspection, inter-rater agreement

## Abstract

Automatic alignment of brain anatomy in a standard space is a key step when processing magnetic resonance imaging for group analyses. Such brain registration is prone to failure, and the results are therefore typically reviewed visually to ensure quality. There is however no standard, validated protocol available to perform this visual quality control (QC). We propose here a standardized QC protocol for brain registration, with minimal training overhead and no required knowledge of brain anatomy. We validated the reliability of three-level QC ratings (OK, Maybe, Fail) across different raters. Nine experts each rated *N* = 100 validation images, and reached moderate to good agreement (kappa from 0.4 to 0.68, average of 0.54 ± 0.08), with the highest agreement for “Fail” images (Dice from 0.67 to 0.93, average of 0.8 ± 0.06). We then recruited volunteers through the Zooniverse crowdsourcing platform, and extracted a consensus panel rating for both the Zooniverse raters (*N* = 41) and the expert raters. The agreement between expert and Zooniverse panels was high (kappa = 0.76). Overall, our protocol achieved a good reliability when performing a two level assessment (Fail vs. OK/Maybe) by an individual rater, or aggregating multiple three-level ratings (OK, Maybe, Fail) from a panel of experts (3 minimum) or non-experts (15 minimum). Our brain registration QC protocol will help standardize QC practices across laboratories, improve the consistency of reporting of QC in publications, and will open the way for QC assessment of large datasets which could be used to train automated QC systems.

## Introduction

Aligning individual anatomy across brains is a key step in the processing of structural magnetic resonance imaging (MRI) for functional MRI (fMRI) studies. This brain registration process allows for comparison of local brain measures and statistics across subjects. A visual quality control (QC) of brain registration is crucial to minimize incorrect data in downstream analyses of fMRI studies. However, no standardized, validated protocol has yet been developed to streamline this QC. Here, we present a standardized procedure for visual QC of brain registration and describe the reliability of QC ratings from both expert raters and a large panel of non-experts recruited through an online citizen science platform^1^.

### Brain Registration

Magnetic resonance imaging is a non-invasive technique that can be applied to study brain structure (sMRI) and function (fMRI). Multiple steps are required to transform raw MRI data to processed images ready for downstream statistical analyses. One critical preprocessing step is brain registration; this involves aligning 3D brain images to a standard stereotaxic space, such as the MNI152/ICBM2009c template (Fonov et al., 2009). State-of-the-art registration procedures use non-linear optimization algorithms such as ANIMAL (Collins and Evans, 1997), DARTEL (Ashburner, 2007), or ANTS (Avants et al., 2009). Dadar et al. (2018) compared five publicly available, widely used brain registration algorithms in medical image analysis and found a failure rate of 16.8 ± 3.13% on their benchmarks. This lack of robustness is mainly due to differences in image quality, shape and cortical topology between individual brains. A visual QC of registered brain images is thus required to ensure good data quality for subsequent analyses.

### Visual QC

The specific focus of the visual QC for sMRI registration depends on the intended use of the data. Voxel-based analysis of brain morphology typically calls for a highly accurate registration, as this step can impact brain tissue segmentation. In contrast, fMRI studies usually rely on larger voxel size and spatial blurring, and are less likely to be affected by small registration errors. To our knowledge, as of yet, there are no standardized criteria for tolerable errors in sMRI registration for fMRI processing pipelines. Many fMRI analytical software packages present users with images to assess the quality of T1 image registration. In one of the most recent packages developed by the community, fMRIprep (Esteban et al., 2019), the registered T1 image is presented across 21 brain slices, along with images for three other processing steps (skull stripping, tissue segmentation, and surface reconstruction), yielding a total of 84 brain slices for visual inspection. Established processing tools like FMRIB Software Library (Jenkinson et al., 2012) or the Statistical Parametric Mapping MATLAB package (Wellcome Centre for Human Neuroimaging, n.d.) also present users with reports that often include more than ten brain slices for visual inspection for each subject. This makes visual inspection tedious and time-consuming. Critically, none of these packages offer guidelines on how to assess the quality of structural brain registration for fMRI studies. Without such guidelines and with a large number of images to review, QC is likely to vary significantly across raters.

### Inter-Rater Agreement

Quality control studies of preprocessed images rarely report inter-rater reliability, and no such study examined brain registration to our knowledge. Pizarro et al. (2016) applied a support vector machine algorithm on visually rated (*N* = 1457 usable/unusable) sMRI data from 5 to 9 investigators who rated the same 630 images, but did not report agreement metrics. White et al. (2018) compared automated QC metrics and manual QC from 6662 sMRI data from 4 different cohorts/sites, merging visual inspection across sites, raters, protocols and scan quality but without presenting agreement statistics. Studies that do report inter-rater agreement mostly focus on issues related to raw MRI images (e.g. signal-to-noise ratio or susceptibility artifacts), head motion (e.g. ghosting or blurring), brain extraction, and tissue segmentation. Inter-rater agreement in these studies is found to vary considerably. For example, Backhausen et al. (2016) reported high agreement for two trained raters who visually inspected the same 88 sMRI, achieving an intra-class correlation of 0.931 for two categories of quality (pass-fail) on issues related to MRI acquisition and head motion. Esteban et al. (2017) reported a kappa of 0.39 between two raters for three quality categories (Exclude/Doubtful/Accept) on 100 images when ratings were based on the quality of the MRI acquisition, head motion, brain extraction and tissue segmentation. Table 1 shows recent (2010 onward) studies reporting inter-rater agreements on visual QC of sMRI for a variety of issues. Only one study, Fonov et al. (2018), included brain registration for visual QC assessment. These authors reported a test-retest Dice similarity of 0.96% from one expert rater who evaluated as pass or fail 1000 images twice, but no inter-rater reliability estimate. Variability in reliability across studies may be due to two types of factors: user- and protocol-related factors. Protocol-related factors (e.g. clarity, levels of rating or training set) can be addressed by multiple iteration and refinement of the protocol. Factors related to the rater (e.g. level of expertise, fatigue, motivation, etc.) are more difficult to constrain or control. One solution to circumvent individual rater variability is to aggregate multiple ratings from a large pool of raters.

**TABLE:** Reported agreement in visual inspection of sMRI data on QC studies.

			Reported visual inspection issue related to				
Study	QC agreement details		MRI Aquisition	Head motion	Brain extraction	Tissue segmentation	Brain registration
Backhausen et al., 2016	Nb. Images	88	Image sharpness, ringing. Contrast to noise ratio (subcortical structures and gray/white matter) and susceptibility artifacts	Ghosting or blurring	N.R	N.R	N.R
	Nb. Raters	2					
	Rating scale	Include/Exclude					
	QC Manual	Supplementary Material					
	Agreement	ICC = 0.93					
Esteban et al., 2017	Nb. Images	100	signal-to-noise ratio. Image contrast and Ringing	Head motion artifacts	Gray/white matter and the pial delineation	Gray–white matter segmentation	N.R
	Nb Raters	2					
	Rating scale	Exclude/Doubtful/Accept					
	QC Manual	N.R					
	Agreement	Cohen’s Kappa = 0 39					
Rosen et al., 2018	Nb. Images	Phasel = l00, Phase2 = 100	N.R	N.R	N.R	N.R	N.R
	Nb. Raters	Phase1 = 2, Phase2 = 3					
	Rating scale	0/1/2					
	QC Manual	N.R					
	Agreement	Phasel = 100%, Phase2 = 85%					
Fonov et al., 2018 (preprint)	Nb. Images	9693 (1000 rated twice)	Effect of noise and image intensity non-uniformity	N.R	N.R	N.R	Incorrect estimates of, translation, scaling in all directions and rotation.
	Nb. Raters	1					
	Rating scale	Accept/Fail					
	QC Manual	Dadar et al., 2018 paper					
	Agreement	intra-rater Dice similarity = 0.96					
Klapwijk et al., 2019	Nb. Images	80	N.R	Ringing	Division between gray/white matter and pial surface	Gray–white matter segmentation	N.R
	Nb. Raters	5					
	Rating scale	Excellent/Good/Doubtful/Failed					
	QC Manual	Supplementary Material					
	Agreement	Reliability = 0.53					

*QC studies since 2010 that uses sMRI and reported their inter/intra-raters agreement (Pizarro et al., 2016; Esteban et al., 2017; Dadar et al., 2018; Fonov et al., 2018; Rosen et al., 2018; Klapwijk et al., 2019). N.R: Not reported.*

### Crowdsourced QC

Crowdsourcing can be used to achieve multiple QC ratings on large collections of images rapidly. Crowdsourcing, as first defined by Howe in 2016, is “*the act of taking a job traditionally performed by a designated agent (usually an employee) and outsourcing it to an undefined, generally large group of people in the form of an open call*” (Howe, 2006). Crowdsourcing can be used in citizen science research projects where a large number of non-specialists take part in the scientific workflow to help researchers (Franzoni and Sauermann, 2014; Simpson et al., 2014). Crowdsourcing labor-intensive tasks across hundreds or thousands of individuals has proven to be effective in a number of citizen science research projects, such as modeling complex protein structures (Khatib et al., 2011), mapping the neural circuitry of the mammalian retina (Kim et al., 2014), and discovering new astronomical objects (Cardamone et al., 2009; Lintott et al., 2009).

In brain imaging, recent work by Keshavan et al. (2018) showed the advantages of using citizen science to rate brain images for issues related to head motion and scanner artifacts. These authors were able to gather 80,000 ratings on slices drawn from 722 brains using a simple web interface. A deep learning algorithm was then trained to predict data quality, based on the gathered rating from citizen science. The deep learning network performed as well as a the specialized algorithm MRIQC (Esteban et al., 2017) for quality control of T1-weighted images. QC of large open access databases like HCP (Glasser et al., 2016), UKbiobank (Alfaro-Almagro et al., 2018) or ABCD (Casey et al., 2018) is challenging and time consuming task if done manually. Using crowdsourced rating could be a key element to rate huge databases and eventually use these ratings to efficiently train a machine learning models to perform QC.

Here, we propose a novel, standardized visual QC protocol for the registration of T1 images by non-experts. We formally assessed protocol reliability, first with “expert” raters familiar with visual inspection of brain registration, and second with a large pool of “non-expert” raters with no specific background in brain imaging. These citizen scientists contributed via the world’s largest online citizen science platform, called Zooniverse (Simpson et al., 2014). Zooniverse enabled the enrollment of more than 2000 volunteers from around the globe, thus enabling the evaluation of consensus between non-expert raters on a large scale. Specific aims and hypotheses of the study were as follows:

1. To establish a QC procedure for MRI brain registration that does not require extensive training or prior knowledge of brain anatomy. Our hypothesis was that such a procedure would help raters achieve more reliable visual QC.
2. To quantify the agreement between a consensus panel composed of non-expert raters and that of experts. Our hypothesis was that the consensus of non-experts would be consistent with experts’ assessments, since the protocol requires no knowledge of brain anatomy.

## Method

### Quality Control Protocol Building

**FIGURE 1:**
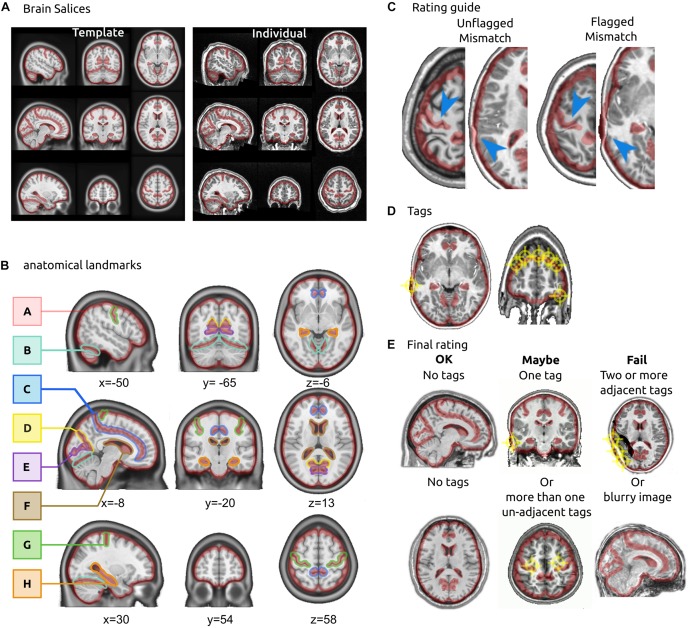
QC protocol for brain registration. **(A)** Brain slices. The rater is presented with two sets of brain slices (3 axial, 3 sagittal and 3 coronal), one of them showing the template in stereotactic space and the other showing an individual T1 brain after registration. In the interface, the two images are superimposed and the rater can flip between them to visually assess the registration. **(B)** Anatomical landmarks. The landmarks for QC included: the outline of the brain (A), tentorium cerebelli (B), cingulate sulcus (C), parieto-cingulate sulcus occipital fissure (D), calcarine fissure (E), the lateral ventricles (F), central sulcus (G) and the hippocampal formation (H) bilaterally. The landmarks were outlined in stereotaxic space. **(C)** Rating guidelines. The boundaries of red landmarks act as “confidence interval” for registration: an area is tagged as a misregistration only if the target structure falls outside the boundaries. **(D)** Tags. Raters put tags on each misregistered brain structure. **(E)** Final rating. A final decision is reached on the quality of registration: an image with no tags is rated OK, one or more non-adjacent tags are rated Maybe, two or more adjacent tags are rated Fail. An image that is excessively blurry is also rated Fail.

The QC protocol was developed iteratively over the past 5 years, with several rounds of feedback from users. Initially, the protocol was used internally in our laboratory (Yassine and Pierre, 2016), and required a visual comparison of T1 slices against a template using the Minctool *register* (Janke and Fonov, 2012). Although the protocol achieved good consistency of ratings between two expert users (kappa = 0.72), it was time consuming and hard to teach. We then switched from an interactive brain viewer to a static mosaic comprised of 9 different slices (3 axial, 3 sagittal, 3 coronal, see Figure 1B), and we highlighted anatomical landmarks using a precomputed mask. These landmarks were selected because we expected all of them to align well in the case of a successful registration, and the precomputed mask served as an objective measure to decide on the severity of a misalignment. We established guidelines on how to rate a registered image on a three-level scale (“OK,” “Maybe,” or “Fail”) using these landmarks. The new protocol limited the need for extensive training for new users and potentially reduced the subjectivity of decision, notably for edge cases. The following sections describe the details and the validation of the final protocol (brain slices, landmarks and rating guidelines).

### Brain Slices

A mosaic view of nine brain slices was extracted from each registered brain. The *x*, *y*, and *z* coordinates, corresponding to axial, coronal and sagittal views, were as follows:

**Table:** 

*x* (sagitai)	*y* (coronal)	*z* (axial)
−50	−65	−6
−8	−20	13
30	54	58

Two images were generated: one using the individual T1 image of a subject, after brain registration, and one using the MNI2009c MRI T1 template averaged from 152 adults after iterative non-linear registration (Fonov et al., 2011), see Figure 1A.

### Anatomical Landmarks

Notable anatomical landmarks included the central sulcus, cingulate sulcus, parieto-occipital fissure, calcarine fissure, tentorium cerebelli, lateral ventricles, bilateral hippocampal formation and the outline of the brain (see Figure 1B). To highlight these landmarks, we hand-drew a red transparent outline inside the brain with the MRIcron drawing tool (Rorden, 2014) using the MNI 2009 gray matter atlas as a reference. For the outline of the brain, we substracted a 4-mm eroded brain mask (MNI2009c release) from a 4-mm dilated brain mask. This process resulted in a roughly 8-mm thick mask centered on the outline of the brain in template space. The landmark boundaries served as the “confidence interval” of acceptable registration. The width of this confidence interval was somewhat arbitrary, but critically helped raters to consistently assess what amount of misregistration was acceptable. The scripts to generate the mosaic brain images with highlighted landmarks have been made available in the GitHub repository^2^.

### Rating Guidelines

We instructed raters to focus on the brain structures within the red anatomical landmarks, comparing the individual brain, after registration, with the MNI 2009c template. The two images were presented superimposed with each other, and raters were able to flip manually or automatically between the individual and the template brain. For a given anatomical landmark, raters were asked to tag any part of the brain structure that fell outside of the anatomical landmark for the individual brain. The template acted as a reference for what the structure looked like, and where it was supposed to be. Figure 1C provides examples of acceptable and unacceptable registration of brain structures within the landmarks. Raters were instructed to click all misregistered brain structures, which resulted in a series of tag spheres with 4 mm radius (Figure 1D). After an image was fully tagged, the overall registration quality was evaluated by the rater as follows:

• “OK” if no tag was reported,
• “Maybe” if one or several regions were tagged, yet no tag spheres overlapped (less than 8 mm apart),
• “Failed” if two tag spheres overlapped, meaning that an extensive brain area (>8 mm) was misregistered. Alternatively, a “Failed” rating was also issued if the entire image was of poor quality due to motion or a ringing artifact (Figure 1E).

### Zooniverse Platform

We used the online citizen science platform Zooniverse (Simpson et al., 2014) as an interface to perform the validation of our QC protocol^3^. Zooniverse offers a web-based infrastructure for researchers to build citizen science projects that require a human visual inspection and possibly recruit a large number of zooniverse volunteers, who are not familiar with neuroimaging and have no formal requirements to participate (Franzoni and Sauermann, 2014; Simpson et al., 2014). Our project, called “Brain match” was developed with the support of the Zooniverse team, to ensure compliance with Zooniverse policies and appropriate task design for an online audience^4^, and the project was also approved by our institutional review board. Note that the raters were considered part of the research team, and not participants of the research project, and thus they were not required to sign an informed consent form. The project underwent a “beta review” phase on zooniverse, where we collected feedback on the clarity and difficulty level of the task. Rating was performed by Zooniverse raters and expert raters. All ratings were performed on the zooniverse platform through the Brain Match dashboard^5^. The rating workflow was the same for the two types of types raters. Note that individuals participating in Zooniverse choose to voluntarily dedicate some of their time to science and thus do not constitute a representative sample of the general population.

### Brain Images Validation and Training Sets

We used a combination of two publicly available datasets, COBRE (Mayer et al., 2013) and ADHD-200 (Bellec et al., 2017), for both the beta and the full launch of the project. These datasets have been made available after anonymization by consortia of research team, each of which received ethics approval at their local institutional review board, as well as informed consent from all participants. Each individual sMRI scan was first corrected for intensity non-uniformities (Sled et al., 1998) and the brain extracted using a region growing algorithm (Park and Lee, 2009). Individual scans were then linearly registered (9 parameters) with the T1 MNI symmetric template (Fonov et al., 2011). The sMRI scans were again corrected for intensity non-uniformities in stereotaxic space, this time restricted to the template brain mask. An individual brain mask was extracted a second time on this improved image (Park and Lee, 2009) and combined with template segmentation priors. An iterative non-linear registration was estimated between the linearly registered sMRI and the template space, restricted to the brain mask (Collins et al., 1994). The processed data were finally converted into mosaics and merged with a mask of anatomical landmarks using in-house scripts. Two expert raters (PB,YB) rated each 954 preprocessed images in ADHD-200, achieving a kappa of 0.72 (substantial agreement) from a random subset of 260 images. The COBRE dataset was rated by YB only.

On Zooniverse, raters were first invited to read a tutorial (Supplementary Figure S2) explaining the protocol, and then completed a QC training session, featuring 15 selected images (5 rated OK, 5 rated Maybe and 5 rated Fail, as rated by YB). Because the COBRE structural images were of higher quality, OK images were selected from COBRE while Maybe and Fail were selected from ADHD-200. For each training image, the rater was first asked to assess the image, and was then able to see the tags and the final ratings by an expert rater (YB).

After completing the training session, raters were presented a series of 100 “open label” cases, and were free to rate as many of these images as they wanted. We chose to present only 100 images in order to ensure we would have many ratings by different raters for each image, within a relatively short time frame. We arbitrary selected a subset of 100 images with a ratio of 35 Fail, 35 Maybe, and 30 OK images based on one expert rater (YB). Once again, the OK images were drawn from COBRE, while the Fail and Maybe were drawn from ADHD-200.

### Raters

More than 2500 volunteers took part in our Brain Match project. They performed approximately 21,600 ratings of individual images over 2 beta-testing phases and two full workflows for a total of 260 registered brain images (see Brain images section). We used a retirement of 40 ratings, which means each image was rated by 40 different Zooniverse raters before being removed from the workflow. Only individuals who rated more than 15 images were kept in the final study. After data cleaning, 41 Zooniverse volunteer raters were kept. The distribution of rating per image showed a mean number of ratings of 21.76 ± 2.75 (see Supplementary Figure S1).

A group of 9 experts raters were also recruited for this study and each asked to rate all of the 100 validation images using the Brain Match interface. They were instructed to first start with the training session and to carefully read the tutorial before starting the main QC workflow. All raters had prior experience with QC of brain registration in the past. Each rater was free to perform the QC task at her pace without any specific direction on how to do it. The process was completed once all ratings were submitted.

Finally, a radiologist was also recruited for the study. He rated the same 100 images using Brain Match interface, also undergoing the training session before the rating process. Although the radiologist had no prior experience in QC of brain registration, that participant had very extensive experience in examining brain images following a standardized protocol, and served as a gold standard about what to expect from a fully compliant rater, trained on QC solely through available online documentation.

### Agreement Statistics

We used Cohen’s kappa (Cohen, 1960) to assess inter-rater reliability across all nine experts (ratings R1–R9). The kappa metric measures the agreement between two raters who rate the same amount of items into *N* mutually exclusives categories. The kappa is based on the difference between the observed agreement (*p*_o_, i.e. the proportion of rated images for which both raters agreed on the category) and the probability of chance or expected agreement (*p*_e_). Kappa (*k*) is computed as follows:

$$k=\frac{p_{o}-p_{e}}{1-p_{e}}$$

In this work we used a weighted kappa metric, which assigns less weight to agreement as categories are further apart (Cohen, 1968). In our QC cases disagreements between OK and Maybe, and between Maybe and Fail count as partial disagreements; disagreements between OK and Fail, however, count as complete disagreements. We used the R package irr (Gamer, 2012) to estimate the weighted kappa and Landis and Koch’s (1977) interpretation of the strength of agreement for κ ≤ 0 = poor, 0.01–0.20 = slight, 0.21–0.40 = fair, 0.41–0.60 = moderate, 0.61–0.80 = substantial, and 0.81–1 = almost perfect.

We also used the Sørensen–Dice coefficient (Dice) to assess the agreement within the rating categories of OK, Maybe and Fail (Sørensen et al., 1948), as follows:

$$D\,S\,C=\frac{2\,\left|X\,⋂Y\right|}{\left|X\right|+\left|Y\right|}$$

where *X* is the set of images rated “OK” by one rater and *Y* is the set of images rated “OK” by a second rater, ⋂ is the intersection between two sets, and |*X*| is the number of images. In plain English, the Dice between two raters for the OK category is the number of images that both raters rated “OK,” divided by the average number of images rated “OK” across the two raters. The same Dice measure was generated as well for “Maybe” and “Fail” images. We interpreted Dice coefficients using the same range of strength of agreement as for the Kappa coefficient (≤0 = poor, 0.01–0.20 = slight, 0.21–0.40 = fair, 0.41–0.60 = moderate, 0.61–0.80 = substantial, and 0.81–1 = almost perfect).

### Consensus Panels

We also evaluated the reliability of QC ratings after pooling several raters into a consensus panel. The panel consensus was generated by counting the number of OK, Maybe and Fail attributed to an image from different raters (number of votes). The category with the highest vote count was selected as the consensus on that specific image for the panel. If there was a tie between 2 or 3 categories, the worst category was selected (Fail < Maybe < OK).

We tested different panel configurations, large and small, for expert and Zooniverse raters separately. Large panels were composed either by all 9 experts (panel Ec) or 41 Zooniverse users (panel Zc). We compared the agreement between Ec and Zc versus each individual expert rater (R1 to R9) as well as the ratings from the radiologist (Ra). For small panel, experts were arbitrarily split into three panels of three raters (panels Ec1, Ec2, and Ec3). The Zooniverse users were also arbitrarily split into two independent consensus panels of roughly equal size (Zc1 and Zc2). We quantified the agreement between small panels, as well as small vs. large panels.

## Results

### Expert Raters Achieved Moderate Agreement, With “Fail” Rating Being the Most Reliable

**FIGURE 2:**
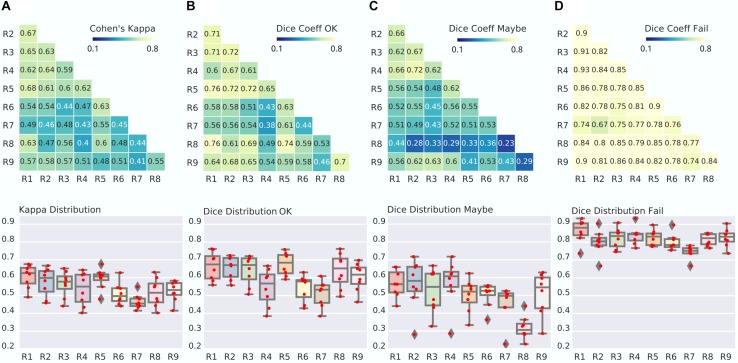
Between-expert agreement. **(A)** Matrix of Kappa agreement between raters (top). Note that R1 to R9 are identification codes for the different expert raters. The distribution of agreement is also presented (bottom). For example, the boxplot for R1 shows the agreement between R1 and R2-R9. **(B–D)** Matrix and distribution for the Dice agreement between raters in the OK **(B)**, Maybe **(C)**, and Fail **(D)** categories.

Kappa agreement between expert raters across the three classes (OK, Maybe, Fail) was moderate to substantial (range 0.4–0.68, average of 0.54 ± 0.08), see Figure 2. However, there were marked differences in agreement across the three rating classes. The highest reliability was for “Fail,” with between-rater Dice agreement ranging from substantial to almost perfect (0.67–0.93, average of 0.8 ± 0.06). The second class in terms of reliability was “OK,” with Dice ranging from fair to strong (0.38–0.76), and the least reliable class was “Maybe,” with Dice agreements ranging from slight to strong (0.23–0.72).

### Large Panel of Experts or Zooniverse Raters Give Convergent, Reliable QC Ratings

**FIGURE 3:**
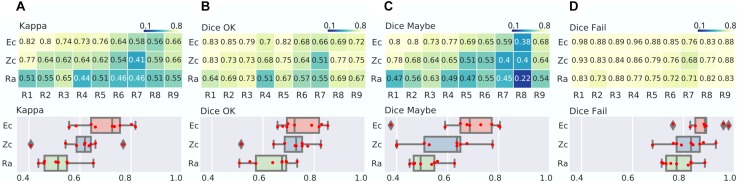
Zooniverse, expert and radiologist agreements. **(A)** Matrix of Kappa agreement between consensus of experts (Ec), zooniverse users (Zc) and radiologist (Ra) raters, in rows, vs. individual experts (R1–R9), in column (top). The distribution of agreement is also presented (bottom). **(B–D)** Matrix and distribution for the Dice agreement in the OK **(B)**, Maybe **(C)**, and Fail **(D)** categories.

We found that the kappa between Ec and individual expert raters was, as expected, improved over comparison between pairs of individual experts, with a range from moderate to strong (0.56–0.82), see Figure 3. As observed before, the Dice scores for Ec were highest in the “Fail” category (almost perfect agreement, range of 0.76–0.98), followed by the “OK” category (from substantial to almost perfect: range 0.66–0.85) and finally “Maybe” (fair to almost perfect, ranging from 0.38 to 0.8). These findings confirmed our previous expert inter-rater analysis, with “Fail” being a reliable rating, “Maybe” being a noisy rating, and “OK” being a moderately reliable rating. When comparing the individual experts with the Zooniverse panel Zc, we only observed a slight decrease in average Kappa compared with the Expert panel (0.61 for Zc vs. 0.7 for Ec), mostly driven by the “Fail” (0.82 for Zc vs. 0.88 for Ec) and “Maybe” (0.58 for Zc vs. 0.68 for Ec) ratings. When directly computing the agreement between the two consensus ranels Ec and Zc, the kappa was substantial (0.76), with almost perfect agreement for “Fail” (Dice 0.9) and “OK” (0.82), and substantial agreement for “Maybe” (0.77), see Figure 3. This comparison demonstrated that aggregating multiple ratings improved the overall quality, and that expert and zooniverse raters converged to similar ratings. The radiologist achieved a level of agreement with panels similar to what was observed with expert raters, and was substantially lower than the agreement between panels. This shows that the QC training material alone was enough for a radiologist to agree with QC experts, but a single user can likely not achieve high quality QC ratings by herself.

### Small Consensus Panels of Expert (*N* = 3) or Zooniverse (*N* = 20) Raters Achieve Reliable QC Ratings

**FIGURE 4:**
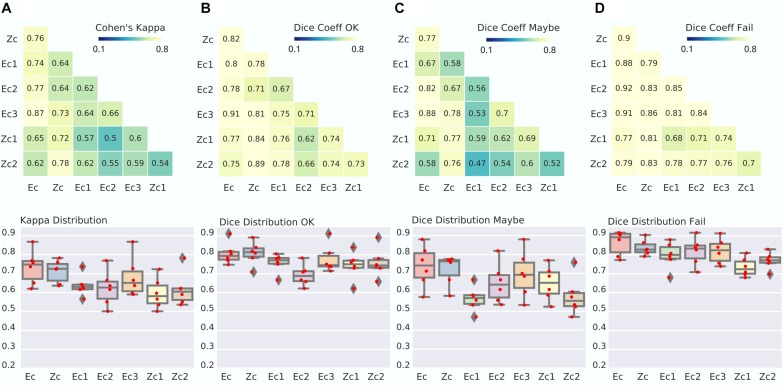
Agreement between small panels of raters for both experts and Zooniverse panels. **(A)** Matrix of Kappa agreement between large panel consensus of experts (Ec), zooniverse users (Zc) and a small panel of expert (Ec1 = 3 rater, Ec2 = 3 rater, Ec3 = 3 rater) and small panel of Zooniverse raters (Zc1 = 20 rater, Zc2 = 21 rater) (top). The distribution of agreement is also presented (bottom). **(B–D)** Dice distribution between group consensus in the OK **(B)**, Maybe **(C)**, and Fail **(D)** categories.

Once we established that large panels of raters lead to high levels of agreements, our next question was to determine whether small panels could also lead to reliable assessments. The small expert panels Ec1-3 reached lower agreement with Zc than the full Ec. Specifically, kappa was 0.64, 0.64, and 0.73 for Ec1 to Ec3 (with respect to Zc), compared to kappa of 0.76 for Ec vs. Zc. Similar observations were done when breaking down the comparison per category with Dice, with a decrease of 5% to 10% in this coefficient (see Figure 4). Comparing small zooniverse panels Zc1-2 with the full expert panel Ec, a slight decrease in reliability was observed, very similar in magnitude with comparisons between Ec1-3 and Zc. The agreements Ec1-3 vs. Zc, as well as Zc1-2 vs. Ec, remained substantial. This suggests that reliable three-level QC assessments can be performed by small panels of three experts (*n* = 3), or moderate panels of zooniverse users, with roughly 20 assessments by image (see Supplementary Figure S1 for distribution).

## Discussion

This project proposes a standardized QC protocol with minimal training overhead and no required knowledge of brain anatomy. Our goal was to quantify the reliability of QC ratings between expert raters, as well as panels of expert or Zooniverse raters. Overall, our results demonstrated that our protocol leads to good reliability across individual expert raters, in particular for “Fail” images, and good reliability across panels of raters (both experts and Zooniverse), even for panels featuring only three experts. To our knowledge, this is the first quantitative assessment of between-rater agreement on QC of brain registration.

### Visual QC

Our protocol was designed to be simple enough that even a rater without brain anatomy knowledge or prior QC experience could generate meaningful ratings. The mosaic view of 9 slices used in our protocol is similar to display images used in fMRI preprocessing tools like MRIQC (Esteban et al., 2017), fMRIPrep (Esteban et al., 2018) or CONN (Whitfield-Gabrieli and Nieto-Castanon, 2012). These QC tools also use an overlay that highlights brain borders or tissues segmentation. Differentiating aspects of our protocol are (1) fewer number of brain slices in the mosaic view, so that raters can more easily examine all presented images and (2) the overlay provides an objective confidence interval to assess the severity of misregistration in key anatomical landmarks. We believe that these two design principles helped reduce the subjectivity of brain registration QC, and increase between-rater agreement, although we did not formally test these hypotheses.

### Inter-Rater Agreement

Table 1 shows that the visual QC agreement reported in recent studies ranged from 0.39 to 0.9. Interestingly, the studies which reached high levels of agreement (0.93–0.96) used ratings with only two levels (ex: pass, fail). Studies with three or more rating levels reported lower agreement scores (0.39–0.85), which were in line with our findings (average of 0.54 for experts). The most challenging rating in our protocol appeared to be the “Maybe” class, featuring mild, spatially limited registration errors. In contrast, good and failed registrations were easily detectable by expert raters. When working with three levels of ratings, the reliability of our protocol is not high enough to work with a single rater. We found that a consensus panel of three experts was sufficient to reach a good level of agreement (average of 0.64), which appears as a minimum panel size to generate high quality QC scores. Aggregating rating between expert or non-expert is a good solution to overcome the variability among human observers on the QC task.

### Crowd Sourced QC

Crowdsourcing QC rating could be one solution to generate high quality QC ratings in big datasets like the UK biobank (Alfaro-Almagro et al., 2018). A recent work from Keshavan et al. (2018) showed that crowdsourced QC ratings on raw brain images can reach the performance of an automated state-of-the-art machine learning QC tool (Esteban et al., 2017). This work relied on a large pool (*N* = 261) of participants, many of whom had prior experience in neuroimaging. We recruited more than 2000 zooniverse non-expert raters, and found that a consensus panel of non-experts with adequate size (about 40 ratings per image) leads to QC ratings of similar quality to a panel of three experts.

### Limitations of the Study

Our study has a number of limitations. First, our protocol is intended to be used with anatomical brain registration in the context of fMRI analyses in volumetric space, rather than surface. Structural brain imaging studies (i.e. cortical thickness) or surface-based fMRI analyses need other protocols that examine more closely fine anatomy and tissue segmentation. Also, our primary use case is large-scale research studies, and not clinical applications. Some clinical applications may require more stringent standards being applied on brain registration. Our protocol was validated with a specific brain registration tool, the CIVET pipeline, and may not be well suited for other algorithms.

Second, we did not control for screen size, screen resolution or fidelity of color representations in our validation, be it with experts or zooniverse individuals. The main use case for our protocol is the review of thousands of brain registration [e.g. in the ABCD sample (Casey et al., 2018)] in a relatively short span of time. The quality control procedure only examines coarse anatomical landmarks, and the required precision of the alignment is on the order of couple of millimeters. For that reason, we think that the characteristics of the screen will not affect significantly between-rater agreement. This is however a potential source of variations which may have decreased the observed agreement, both between experts and zooniverse raters.

Third, The success rate of our registration tool varies widely as a function of the imaging protocol. The Cobre dataset has almost only OK registration, while the ADHD has a lot of Maybe and some Fail. So we decided to mix two dataset, in order to assemble representative examples of the three classes. This may influence the results by increasing the potential agreement, if subjects learned to recognize which datasets the examples originated from.

Fourth, our choice on the number of rated images (*N* = 100) was selected arbitrarily. We checked the appropriateness of that choice by assessing the minimum number of rated cases with a three-choice decision using the R package “irr” (Gamer, 2012), that uses the minimum sample size estimation formula from Flack et al. (1988). We estimated the minimum sample size under the following scenario. The vector of marginal probability was given by rates for the 3 categories, OK = 0.3, Maybe = 0.35 and Fail = 0.35. These marginal probabilities were decided by our team when designing the dataset, based on an initial QC assessment performed by YB and PB. The value of kappa under the null hypothesis was set equal to 0.5 (k0 = 0.5) – i.e. we want to demonstrate an improvement over a baseline κ of 0.5. The true kappa statistic estimated between two expert was set equal to 0.72 (k1 = 0.72), as was observed in our sample. The type I error test was set equal to 0.05 (α = 0.05). The desired power to detect the difference between the true kappa and the null kappa was investigated at 0.8 and 0.9, separately. The required number of ratings was estimated at *N* = 54 for a power of 0.8, and *N* = 72 for a power of 0.9. In our case, the number of images rated per expert was *N* = 100, which is more than required by the power analysis.

Fifth, We were unable to assess to what degree this protocol improves or not over current best practices in the fMRI community, in the absence of other standardized protocols available for comparison. We still produced preliminary evidence while developing the current protocol. During the beta phase of our project, we tested the agreement between consensus of Zooniverse raters and experts raters (on 29 images). The protocol used during that phase was different from the actual one. In particular, we did not instruct raters on how to take the final decision on the quality of registration (Figures 1C–E), and we did not offer a training set. The kappa measure between consensus Zooniverse raters and an expert during phase 1 was 0.34, by contrast with 0.61 using the current protocol. We regard these results as preliminary evidence that our protocol improves over our previous iteration. These results are to be interpreted with caution, as the number of images rated was low and we used only one expert rating. Note that the feedback received by beta testers helped us identify the importance of steps described in Figures 1C–E, and we suspect that protocols that do not include such detailed explanation have poor reliability. But we did not attempt to demonstrate this formally within the scope of the present study.

Finally, our protocol is missing an evaluation of another key registration step, i.e. alignment between functional images and the structural scan (Calhoun et al., 2017). We are currently working on an extension of our protocol for functional registration.

### Future Work: Impact of QC on Downstream Analyses

Despite the ubiquity of visual brain registration QC in the neuroimaging research community, the impact of visual QC of brain registration on statistical analyses remains poorly characterized. Gilmore et al. (2019) used a multi-site dataset of structural MRI images with different age ranges to show how automated image quality metrics impacted regional gray matter volumes and their relationship with age. Ducharme et al. (2016) showed a significant impact of visual QC on the estimation of cortical trajectories. They demonstrated that, when omitting to discard subjects that did not pass QC, the developmental trajectory of cortical thickness followed a quadratic or cubic trend. By contrast, after filtering those subjects, the trajectory followed a linear trend. Standardizing the QC protocol will allow different laboratories to join their effort of rating and open up new opportunities to systematically investigate the impact of visual QC on the relationship between the brain and various phenotypes. This represents an important area of future work for brain registration.

## Conclusion

Our QC protocol is the first reliable visual protocol for brain registration in fMRI studies. The protocol is easy to implement and requires minimum training effort. This protocol demonstrates a good reliability when performing a two level assessment (Fail vs. OK/Maybe) by an individual rater, or aggregating multiple three-level ratings (OK, Maybe, Fail) from a panel of experts (3 minimum) or non-experts (15 minimum). The images necessary to apply the protocol can be generated using an open-source tool, called dashQC_fmri (Urchs et al., 2018) and a live version can be tested on this link https://simexp.github.io/dashQC_BrainMatch/index.html. We hope this new protocol will help standardize the evaluation and reporting of brain registration in the fMRI community. This standardization effort will also enable the generation of high quality QC ratings on large amounts of data, which will in turn allow to train machine learning models to automatically perform brain registration QC, alleviating the need for visual review.

## Data Availability Statement

The datasets generated for this study are available on request to the corresponding author.

## Ethics Statement

The studies involving human participants were reviewed and approved by the Comité d’éthique de la recherche vieillissement-neuroimagerie. Written informed consent for participation was not required for this study in accordance with the national legislation and the institutional requirements.

## Author Contributions

YB, AB, SU, HS, and PB contributed to the conception, revision, and design of the QC protocol. YB, SU, and PB generated and reviewed code for the study. YB, JA, and SU developed the QC interface. TO performed the rating as gold standard. All authors contributed to the manuscript revision, read, and approved the submitted version.

## Conflict of Interest

The authors declare that the research was conducted in the absence of any commercial or financial relationships that could be construed as a potential conflict of interest.

## Footnotes

1 www.zooniverse.org

2 https://github.com/SIMEXP/brain_match

3 https://www.zooniverse.org/projects/simexp/brain-match

4 https://www.zooniverse.org/lab-policies

5 https://www.zooniverse.org/projects/simexp/brain-match
