# Supplementary material for: A Standardized Protocol for Efficient and Reliable Quality Control of Brain Registration in Functional MRI Studies
Source: Front Neuroinform. 2020 Feb 28;14:7. doi: 10.3389/fninf.2020.00007 (PMC7059806; doi:10.3389/fninf.2020.00007)
Supplement: Supplementary file 1 [file Data_Sheet_1.PDF]

Supplementary Material

1 SUPPLEMENTARY FIGURES

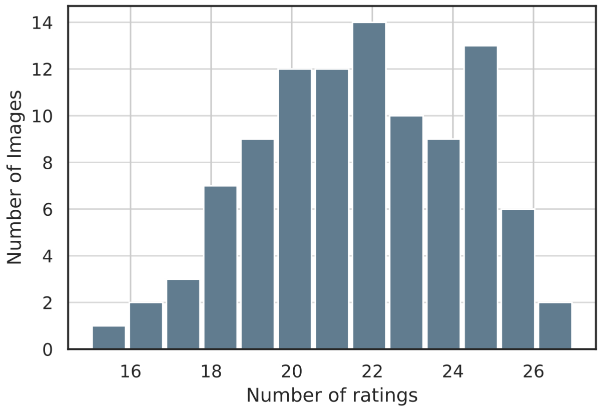

Figure S1. Distribution of images as a function of the number of ratings (mean of 21.76 ± 2.75, range 15 to 27)

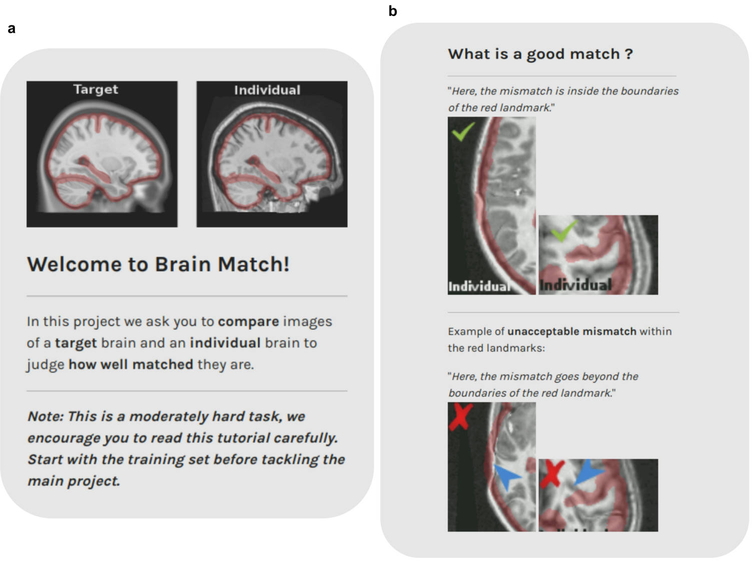

Figure S2. **a:** A tutorial popup when first accessing the brain match interface showing the main goal of the task. **b:** A panel of annotated examples of what is a good and bad match between highlighted brain area. More examples follows on how to tag images and how to decide the final rating of each image (for more detailed tutorial visit https://www.zooniverse.org/projects/simexp/brain-match)
